# Exploring the usability of the COM-B model and Theoretical Domains Framework (TDF) to define the helpers of and hindrances to evidence-based practice in midwifery

**DOI:** 10.1186/s43058-020-00100-x

**Published:** 2021-01-12

**Authors:** Annemarie De Leo, Sara Bayes, Dianne Bloxsome, Janice Butt

**Affiliations:** 1 Edith Cowan University, 270 Joondalup Drive, Perth, WA Australia; 2 King Edward Memorial Hospital, Perth, WA Australia

## Abstract

**Background:**

Despite the advancement of scientific research in the field of maternity care, midwives face challenges translating latest evidence into evidence-based practice (EBP) and express reticence towards leading practice change in clinical areas. This study aimed to explore midwifery leaders’ views on what factors help or hinder midwives’ efforts to translate latest evidence into everyday practice and consider them in relation to both the Capability, Opportunity, Motivation and Behaviour (COM-B) model and Theoretical Domains Framework (TDF).

**Methods:**

This qualitative study formed part of a larger action research (AR) project that was designed to improve midwives’ EBP implementation capability. Data were obtained from eight Western Australian midwifery leaders who were employed in either managerial or executive positions within their organisation. Five midwives attended a focus group workshop and three opted for face-to-face interviews. Thematic analysis was used to code the transcribed data and group alike findings into sub-categories, which were collapsed to four major categories and one overarching core finding. These were mapped to a matrix combining the COM-B and TDF to establish the usability of these tools in midwifery contexts.

**Results:**

Four major categories were developed from the data collected in this study. Three reported the hindrances midwives’ experienced when trying to initiate new EBPs: ‘For midwives, medical opposition and workplace culture are the biggest challenges’, ‘Fear can stop change: it’s personal for midwives’ and ‘Midwives are tired of fighting the battle for EBP; they need knowledge and the confidence to bring about practice change.’ The other major category highlighted factors midwives’ considered helpers of EBP: ‘Having stakeholder buy-in and strong midwifery leadership is a huge advantage.’ When mapped to the TDF and COM-B, these findings provided valuable insight into the helpers of and hindrances to evidence-based practice in midwifery.

**Conclusion:**

Midwives are motivated to initiate evidence-based change yet have limited knowledge of implementation processes or the confidence to lead practice change. Factors such as inter-disciplinary buy-in, clear instruction for midwives and support from midwifery leaders were considered beneficial to implementing practice change in clinical areas. The TDF when used in combination with the COM-B was deemed useful to midwives wanting to lead practice change projects in clinical areas.

Contributions to the literature

- The benefits of evidence-based practice (EBP) in healthcare are well reported; however, low rates of adoption and inconsistent use of latest evidence in clinical areas remains challenging for midwives.
- We found the COM-B and TDF in combination were useful for exploring the hindrances and helpers of EBP in midwifery practice and would be beneficial to midwives wanting to initiative evidence-based change in clinical areas.
- The findings of this study provide empirical evidence about the helpers and hindrances of EBP in midwifery, highlighting the usability of implementation science (IS) tools and change theories to address the evidence-to-practice gap problem in maternity care.

## Background

The benefits of adopting evidence-based practice (EBP) in healthcare are well reported in the literature [7, 24]. However, after more than two decades of implementation science (IS) research and the development of over 60 implementation theories, models and frameworks, the evidence-to-practice gap remains a problem in healthcare [8].

**Fig. 1:**
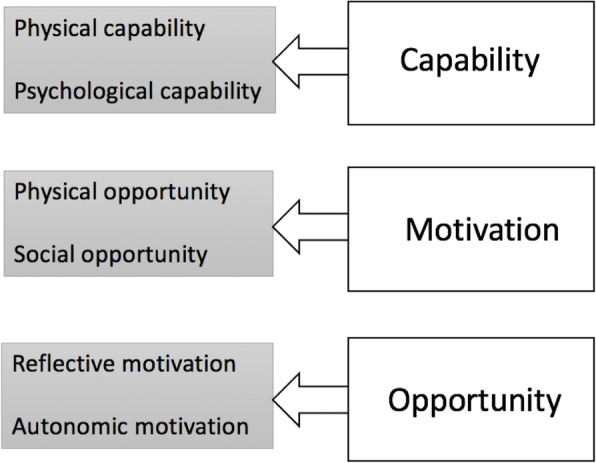
The domains of the COM-B, adapted from Keyworth et al. [13]

The implementation of strategies that target behaviour change is recognised to be more effective when implementation theory is used, in comparison to those that lack a philosophical approach [9, 10]. This is evident in midwifery, where the use of theory has been known to contribute to better understanding evidence implementation processes and projects aimed at behaviour modification [6]. One such theoretical framework, the ‘Capability, Opportunity, Motivation and Behaviour’ (COM-B) model, also recognised as the ‘Behaviour Change Wheel’ (BCW), is widely used to contextualise individual-level change and the underlying determinants of what must occur in order to achieve organisational change [19]. The key premise of the COM-B lies in understanding how *capabilities* (an individual’s capacity to engage in behaviour modifications), *opportunity* (factors in the environment that influence individual behaviours) and *motivation* (the willingness to change) can be used to generate actions that positively impact interventions targeted at behaviour change [11]. These three domains are further divided into six sub-domains (Fig. 1) that capture the factors known to influence an individual’s capacity to adopt new behaviours [13].

Context assessment frameworks derived from IS research may also provide valuable insight into the challenges of implementing EBP. The Theoretical Domains Framework (TDF) builds on the systems identified in the COM-B to further uncover the underlying barriers and facilitators of evidence-based change [4]. Comprising 14 domains, the TDF provides a comprehensive grouping of the overlapping constructs within behavioural theories [17]. These constructs expand on the 14 domains, providing clinicians with an explanation of the domains and their definition (Table 1).

**Table 1:**
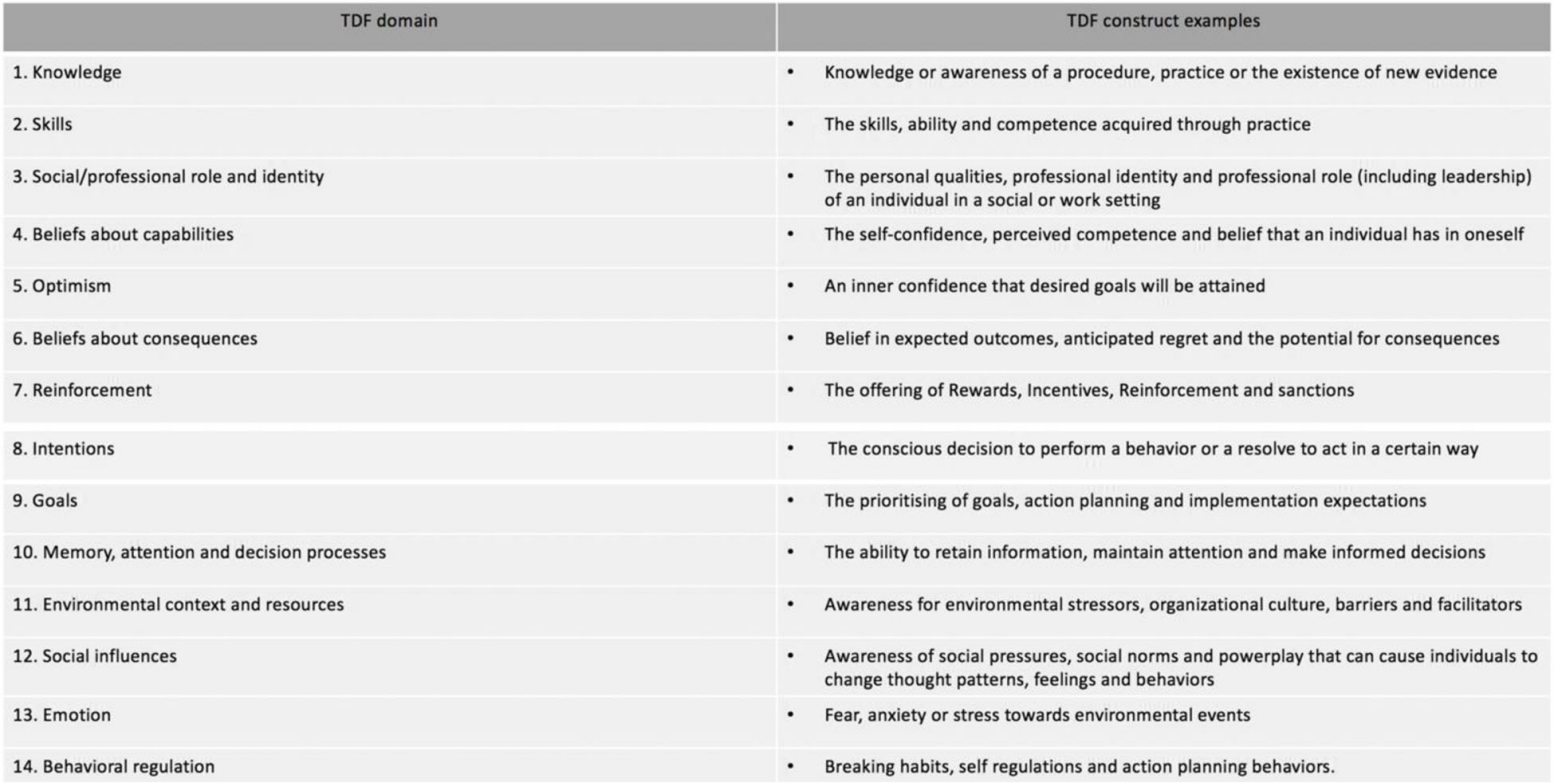
The Theoretical Domains Framework with exemplar constructs, adapted from Cane et al. [4]

Both the COM-B and TDF have previously been used in midwifery contexts to better understand the various behaviours of women during pregnancy [5]. However, there have been no advances on the usability of these tools with regard to EBP or the implementation of evidence-based behaviours in clinical areas.

Implementation science knowledge is not commonly taught in midwifery education and although literature on the topic continues to inform midwives of the evidence-to-practice problem, it fails to provide clear direction on how to facilitate practice change activities [20]. McLellan et al. [17] report on midwives’ perceptions of the barriers to enacting EBP (such as increasingly heavy workloads, difficulty accessing appropriate training and lack of support), acknowledging the absence of interventions designed to support midwives’ address the barriers to enacting evidence-based behaviours in practice. There is an established body of literature on the barriers and facilitators of EBP in maternity care [23], although limited literature exists on midwives use of IS resources to facilitate the process [2]. The purpose of this study was to address this uncertainty by exploring midwife leaders’ experiences of implementing EBP and testing the usability of the COM-B and TDF in midwifery contexts.

## Methods

Action research (AR) was the underpinning methodology selected for this study. First coined by Lewin [15], AR is described as a methodology explicitly founded on a partnership approach to problem-solving [12]. Action research involves the simultaneous achievement of positive actions through four distinct stages: planning, action, observation and evaluation [22]. Collaboration is fundamental throughout each stage and participants are encouraged to both partake and contribute to the research process [18].

### Study design

This study formed the first phase of a broader AR project that was designed to improve midwives’ capability to lead practice change projects in clinical areas. Qualitative description (QD) was employed to gain insight into midwives’ information, tools, skills and support needs with regard to introducing new evidence-based interventions or practices in the workplace. Qualitative description is an approach widely used in healthcare where activities or individual experiences provide insight into a poorly understood phenomenon [14].

### Population

Participants were purposefully selected for their extensive experience in midwifery leadership roles in which they had either overseen or led practice change initiatives. Recruitment was via an online invitation. Six Directors of Midwifery from six maternity service sites in Western Australia (WA) were invited to nominate 1–2 midwives holding leadership positions within their organisation. Eight midwife leaders were nominated, and all consented to participate.

### Data collection

Two methods of data collection were employed for this study: one focus group discussion comprising five participants and three face-to-face interviews for the remaining participants. Both methods of data collection were guided by four discrete discussion points focusing on participants’ experiences of initiating evidence-based change, the information and tools midwives consider important to implementing new EBPs, the factors that should be included in an evidence implementation resource for midwives (if any), and how these should be packaged and presented (if at all) to best suit the needs of diligent midwives working in clinical areas (Table 2). The focus group workshop was facilitated over 3 h and the semi-structured interviews each lasted approximately 60 min. All discussions were audio-recorded and additional field notes taken. All participants were ascribed pseudonyms.

**Table 2:** Focus group and face-to-face interview discussion points

• What are your experiences of implementing evidence-based change in your organisation? • What information or tools should be considered when developing an evidence implementation resource for midwives? • What other factors should be considered when implementing new EBPs in clinical settings? • How should this resource be packaged to best suit the needs of busy midwives working in clinical areas?	

### Ethics

Approval to conduct the study was granted by the Human Research and Ethics Committee at Edith Cowan University on 30 January, 2019. No risks to the participants or the researchers were anticipated, and none eventuated.

### Data analysis

**Fig. 2:**
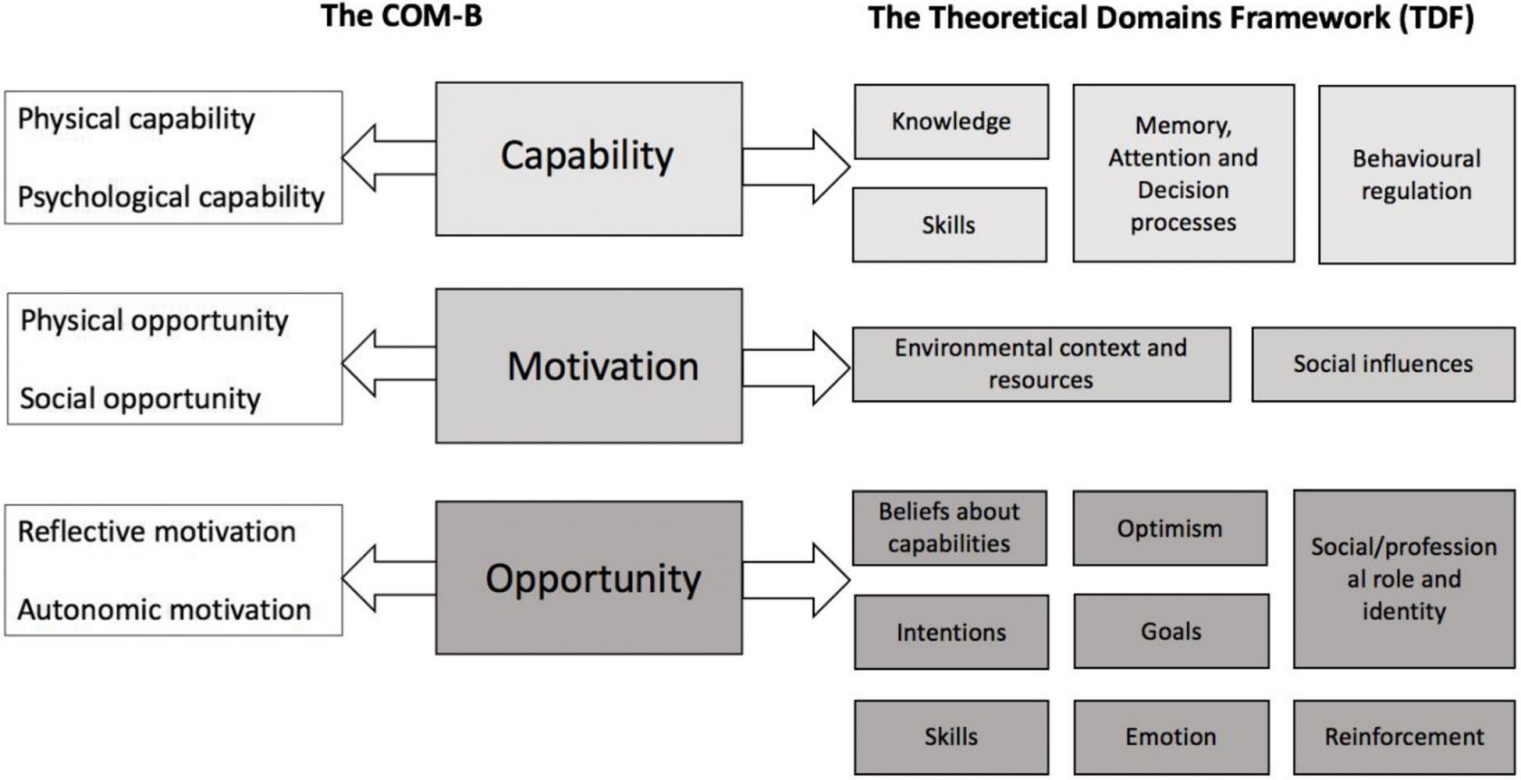
The COM-B and TDF matrix [4, 19]

Consistent with the QD approach, audio-recordings and field notes from the focus group workshop and face-to-face interviews were transcribed and coded through a process of parsimonious thematic analysis as described by Braun and Clarke [3]. This comprised generating initial codes that were then collated into meaningful sub-categories. These sub-categories were collapsed into major categories and finally, one overarching core finding was developed. Content analysis was employed to identify and group alike codes together, reducing the volume of text collected while staying true to the transcripts. The major categories and their constituent data were mapped to a matrix comprising the COM-B and the TDF [4, 19] (Fig. 2). This process was conducted by authors 1, 2 and 3, both independently and together, through an iterative course until consensus was achieved.

## Results

There was unanimous agreement by all eight participants that midwives are passionate about EBP yet reticent towards leading change. According to participants, the reasoning behind this was midwives’ limited knowledge of implementation processes, medical opposition and a perceived lack of confidence to lead practice change activities. Seventy-two codes were grouped into initial sub-categories, which were collapsed to form four major categories. These major categories were then further collapsed into one core finding. Three major categories were identified as hindrances of EBP and one represented helping factors (Table 3).

**Table 3:**
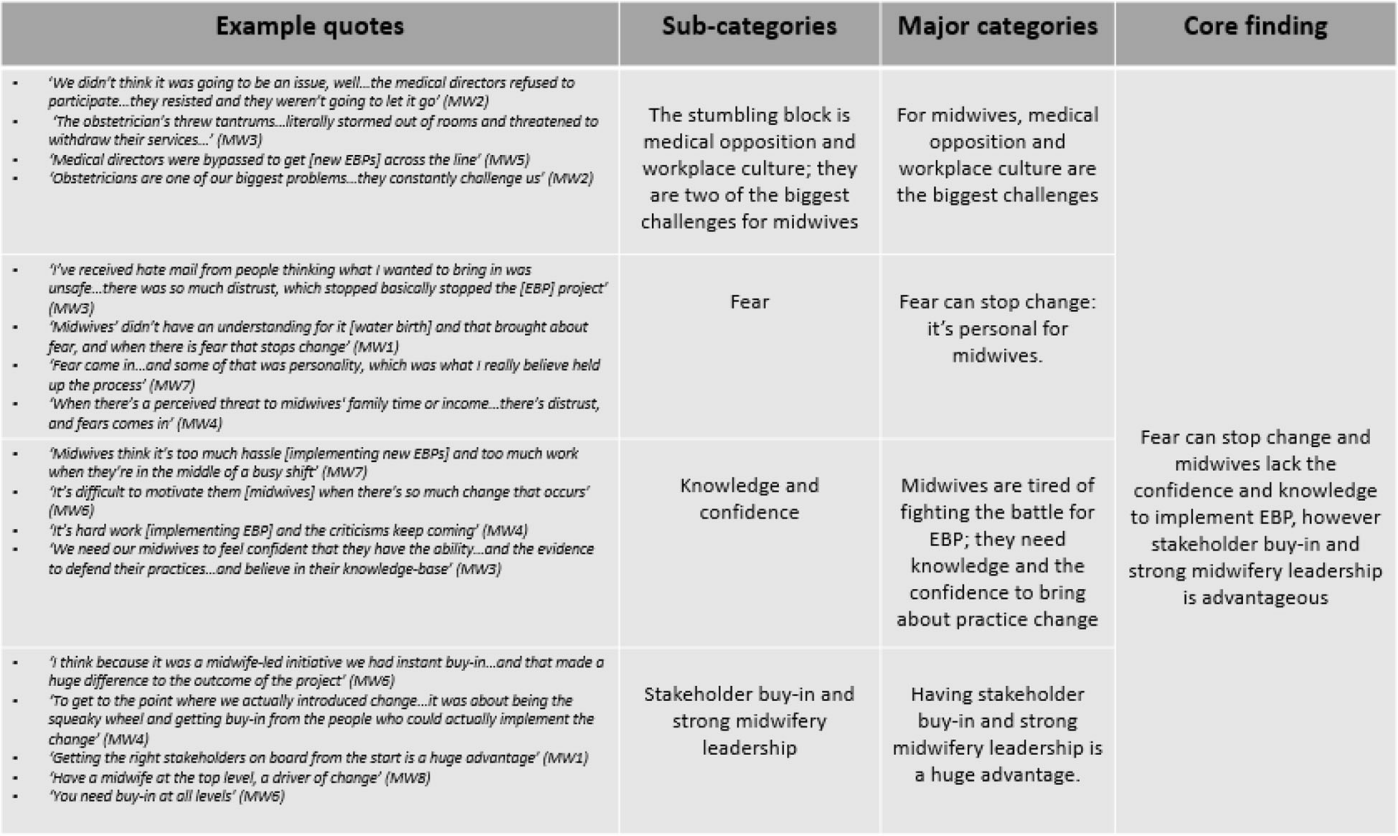
Example of the findings, highlighting the helpers of and hindrances to EBP

The outcomes of the analysis were then mapped to a matrix that combined the COM-B with the 14 domains of the TDF. It was anticipated that combining the behaviour-focused COM-B model with the TDF would result in a better understanding of what needs to occur for midwives to successfully implement new EBPs in clinical areas.

### Capability: physical and social

Within the *capability* system of the COM-B, three of the TDF domains (knowledge, skills and beliefs about capabilities) were described by participants, who described both physical and psychological capabilities (and limitations) towards implementing EBP. The majority of participants recognised time restrictions during work hours limited their capacity to initiate and sustain practice change activities, given their heavy workloads and rostering systems. One midwife voiced ‘change takes time and you also need to be present with women…you’ve got to manage both and that’s sometimes not easy’ (MW7). All participants acknowledged the challenges of implementing evidence-based change during work hours, with the consensus being ‘if you’re not physically present, change just doesn’t happen’ (MW2). Similarly, MW7 shared her experience of trying to introduce bedside clinical handover in her workplace, stating midwives ‘seemed keen, but there was an unspoken resistance…and if I wasn’t physically present at handover time…it just didn’t happen.’ MW2 agreed as she recollected the effort required when she tried to introduce ‘peanut balls’ to birth suite midwives at her workplace: ‘we had all the evidence to support this equipment, paid for our midwives to attend workshops…and demonstrated the peanut balls increased our vaginal birth rates significantly.’ All participants described the resistance they experienced from staff, collectively voicing frustration at the general pace of change in clinical areas. This was captured in a comment by MW3, who said ‘I still see the peanut balls put in the cupboard three months on and question is change actually happening here?...I have to be onto it, physically checking the rooms to make sure the balls are being used, and this equipment is evidence-based.’

With regard to midwives’ psychological capabilities, participants expressed midwives generally express reticence towards leading practice change, as MW6 voiced ‘we want to do it [implement new EBPs]…but can’t do it now.’ MW1 agreed, suggesting ‘it’s difficult…our midwives need to feel capable…that they have the ability and evidence to support new practices…but they don’t believe in themselves’. MW6 concurred, commenting that ‘midwives think it’s too much hassle [implementing practice change] and too much work when they’re in the middle of a busy shift.’ She later continued ‘it’s difficult to motivate them [midwives] when there’s so much change that occurs.’ Together, these sentiments aligned with the TDF domains, which enabled further exploration of midwives’ capabilities in relation to their knowledge, skillsets and behaviours towards implementing EBP.

### Opportunity: physical and social

Two of the TDF domains (environmental context and resources and social influences) were identified in the codes and sub-categories as being suited to the *opportunity* component of the COM-B. This was further explored with regard to the *opportunity* sub-domains: physical opportunity and social opportunity. Participants articulated numerous social and organisational hindrances that hindered midwives’ efforts to introduce new EBPs. Social influences were explored by MW5 who recalled conversations with one midwife who said ‘that sounds like a great idea, and in a perfect world if I didn’t need sleep, have my family and need to pay the bills I would [initiate practice change]…let’s wait till next year.’ Another midwife voiced that ‘midwives feel a lot of pressure to conform to organisational norms, and what’s right for the organisation does not always reflect our midwifery philosophy’ (MW2). Physically, participants expressed several hindrances to EBP, which aligned with the TDF domains that were mapped to *opportunity* in the COM-B. For example, MW3 suggested the limited resources at her workplace were the biggest physical hindrance to EBP: ‘We don’t have access to reliable WIFI or a space that’s dedicated to midwives’ working on quality improvement projects.’ MW7 agreed, commenting ‘the success of change efforts largely depends on the resources you’ve got and the people available to embed practice change initiatives into work environments.’ These discussions led to participants identifying other various contextual factors that influenced change efforts, with workplace culture identified as ‘one of our biggest problems’ (MW3). This was exemplified by MW6, who shared her experience of trying to introduce sterile water injections as an option for pain relief in her organisation’s birth suites. She experienced ‘rumour mongering’ from ‘people working in the service who did not trust the evidence…it was a cultural thing.’ This was made more challenging by staff saying ‘I want to do it, but I’m too busy to make it happen [initiate practice change].’ MW1 shared a similar experience when she tried to introduce water birth facilities at her workplace, disclosing ‘I’ve received hate mail from people thinking what I wanted to bring in was unsafe…there was so much distrust for a practice that is essentially evidence-based.’

The resistance experienced by all participants not only delayed the prospect of initiating evidence-based change but also lengthened the time it took to sustain new practices. This resulted in inconsistency in both the uptake and longevity of practice change projects.

### Motivation: reflective and autonomic

When mapped to the COM-B, the TDF domains identified in this system included behavioural regulation, beliefs about consequences, social/professional role and identity, emotion, optimism and reinforcement. Significantly, participants expressed reticence towards practice change. This led to aversion by some midwives, who reflected upon the problems and challenges associated with initiating evidence-based change. MW7 recalled a conversation with one of her midwives, who questioned ‘why are we changing things again?...we’re busy enough already…I just don’t have the time now’ (MW7). Participants also reported that many midwives were driven by automatic (emotional) responses to change, which often related to their personal views towards EBP and how practice change would affect their workload and professional responsibilities. One midwife quoted ‘I didn’t say I don’t believe in it [EBP], I just want to know how it’s going to affect my workload and income?’ (MW4). Domain 13 of the TDF (emotion) provided a platform for participants’ descriptions of stress, fatigue and anxiety towards introducing new EBPs as reflected in a comment by MW8: ‘midwives are worn down, they’re tired and this affects their psyche.’ Another significant finding reported was that midwives’ fear initiating change. Largely, this was attributed to the isolation and intimidation midwives experience when trying to introduce evidence-based change. For example, MW5 voiced ‘I’ve felt physically intimidated by colleagues who refused to accept the practice I was trying to introduce…I’ve shed a lot of tears….’ The TDF proved valuable in deconstructing this further, highlighting many midwives feel reticent towards practice change because they have observed the challenges other midwives’ experience when trying to introduce new practices into work environments.

Midwives’ motivation to implement a practice change was explored through domain seven of the TDF (reinforcement), which also mapped to *motivation* in the COM-B. MW6 suggested ‘there’s not enough incentive to motivate midwives to change…mostly because there are so many changes occurring…it’s difficult to motivate them [midwives] unless you’re offering some kind of reward…and we can’t afford that’ (MW8). No participants reported reinforcement techniques as articulated within the constructs of reinforcement (TDF domain 7).

Feelings of optimism (TDF domain 5) resonated in the views shared by most participants, as exemplified by MW8, who said ‘I think they’ve (midwives) done amazing with embracing change…we can’t lose sight of that’. The constructs within this domain also reflect the social/professional role and identity of midwives (TDF domain 3), which captures the professional responsibility of midwives to lead change initiatives in maternity care settings. MW3 reflected on these issues, suggesting ‘when we lead initiatives we get things done…and we don’t do things individually, you need buy-in at all levels…and we have to be united…all in or all out’. Arguably, MW4 captured the essence of *motivation* in her thinking ‘to get to the point where we could actually introduce change, it was about being the squeaky wheel and getting buy-in from the people who could make a difference.’

## Discussion

This study aimed to establish midwives’ views on the helpers and hindrances of EBP and tested the suitability of the COM-B and TDF to further explore the underlying factors that contribute to the timely adoption of EBP in clinical areas. This was achieved, although significantly none of the participants had previously considered or used IS tools to support their implementation efforts. This perhaps reflects the near absence of midwifery research relating to IS and offers an explanation for the persistent evidence-to-practice gap in maternity services. The findings of this study resonate with those reported by Bayes et al. [2], who acknowledge the limited use of IS tools in midwifery despite their reported value in other healthcare contexts. This view is also consistent with literature reporting on the usability of the COM-B and TDF outside the discipline of midwifery [1, 16].

In regard to this study, only two of the TDF domains were not identified in the findings: intentions and goals (TDF domains 8 and 9). This offers some insight into why participants experienced the challenges they reported and may provide direction for future implementation processes in midwifery. Although all participants set broad goals to implement evidence-based change, none specifically spoke of the processes they used to plan, implement, evaluate and sustain their implementation efforts. We do not assume these steps were not undertaken, rather highlight the need for midwives to consider goal setting and action-planning when implementing EBP. Although ongoing audit and evaluation were reported by two of our participants, none articulated how they intended to address behavioural change or recognised the value of incorporating IS processes in their implementation projects.

We considered this mapping exercise beneficial for diagnosing the underlying factors that both help and hinder midwives’ efforts to lead practice change initiatives in clinical areas. When used in combination, the COM-B and TDF were capable of highlighting where midwives must focus their attention to successfully lead practice change initiatives, while providing insight into what implementation strategies may be needed to address the individual and organisational hindrances of evidence-based change. In this context, the Expert Recommendations for Implementing Change (ERIC) may prove a useful tool for midwives planning to initiate practice change in clinical areas [24]. The ERIC tool comprises a compilation of 68 implementation strategies that provide a foundation for constructing intervention strategies aimed to improve to the outcomes of quality improvement projects (for example, education, training and environmental restructuring). These are multi-dimensional and useful for targeting change innovations at both individual and organisational levels [21]. Although not context specific, the ERIC compilation may be of use to midwives wanting to target intervention strategies specific to the implementation helpers and hindrances explored in this study.

### Limitations

This study must be considered within the context in which it was conducted. Although the sample provided sufficient data to generate significant findings in this study, the participants represented a relatively small portion of experienced midwifery leaders from the WA public health sector and we may have benefited from the inclusion of practicing midwives. Thus, it is possible the findings of this study may not reflect the wider implementation issues of practicing midwives in all maternity care contexts.

## Conclusions

The findings of our study are essentially that midwives feel hindered to implement EBP for the following reasons: fear, lack of IS knowledge and confidence to lead practice change, workplace culture and medical opposition. Comparatively, having stakeholder buy-in and strong midwifery leadership were identified as helping factors for midwives wanting to initiate new EBPs in clinical areas. Employing the COM-B and TDF (in combination) to diagnose these hindrances and helpers proved beneficial in exposing the areas of focus midwives must direct their attention to address the challenges associated with initiating practice change activities. The findings of this study also provide valuable insight into how midwives might develop intervention strategies specific to the implementation issues midwives experience in clinical areas. Ultimately, this may lead to the development of evidence implementation processes designed to support midwives’ efforts to lead evidence-based change and address the persistent evidence-to-practice gap in maternity services. Midwives are key stakeholders in this venture and thus should be consulted in future research designed to improve the implementation of EBP in maternity services.

## Data Availability

The data sets during and/or analysed during the current study are available from the corresponding author on reasonable request.

## Abbreviations

IS: Implementation science
EBP: Evidence-based practice
COM-B: ‘Capability, Opportunity, Motivation and Behaviour’
TDF: Theoretical Domains Framework
